# Increased Prevalence of Advanced Adenomas in Younger Individuals Undergoing Early Screening Colonoscopy Due to a Family History

**DOI:** 10.1016/j.gastha.2026.100972

**Published:** 2026-04-16

**Authors:** Jin Yun, Douglas Hanes, Branden Tarlow

**Affiliations:** 1 Department of Medicine, Providence Portland Medical Center, Portland, Oregon; 2 Division of Gastroenterology, The Oregon Clinic East, Portland, Oregon

**Keywords:** Colorectal Cancer, Colorectal Cancer Screening, Advanced Adenomas, Family History of Advanced Adenomas, Colonoscopy

## Abstract

**Background and Aims:**

Current guidelines recommend patients with a first-degree relative with advanced adenomas (AAs) identified <60 years old should initiate screening colonoscopy at age 40 or 10 years prior to their relative. Data on the prevalence of AAs and colorectal cancer in these patients, especially younger individuals, are lacking. This study determined the prevalence of AAs and colorectal cancer in younger individuals undergoing early screening colonoscopy due to a family history of advanced polyps.

**Methods:**

A retrospective case-control study performed at a large community practice identified 409 patients who underwent screening colonoscopy due to a first-degree relative with advanced polyps <60 years old (“FH” [family history]) and 11,441 patients without a family history (“control”) who underwent a colonoscopy for abdominal pain or bowel changes between 2015 and 2023. Patients were divided into 3 age groups of 20–39, 40–45, and 45–49 years old for statistical analysis. The primary outcome was the detection of AAs.

**Results:**

The prevalence of AAs was greater in the FH group (n = 40) compared to the control group (n = 682) (9.8% vs 6.0%; *P* < .001). There was nearly a 3-fold increased risk of AAs in 20- to 39-year-old patients in the FH group compared to the control in the same age group (odds ratio 2.66 [95% confidence interval, 1.17–5.29]).

**Conclusion:**

A family history of AA was associated with an increased prevalence of advanced polyps in younger adults compared to a control population. Prospective research is needed to further evaluate the prevalence of AA in this population.

## Introduction

Recent epidemiologic data have shown a concerning rise in colorectal cancer (CRC) among individuals under 50 years of age, which has prompted increased emphasis on early detection strategies in younger populations. Advanced adenomas (AAs) are substantially more prevalent than CRC and are increasingly identified in younger adults during routine screening and diagnostic evaluations.^1^ The US Multi-Society guidelines recommend patients with a first-degree relative (FDR) with AA identified before age 60 initiate screening colonoscopy at age 40 or 10 years prior to their relative. This recommendation is based on indirect evidence from studies that have notably excluded younger populations, those most impacted by the current screening strategy.^2^, ^3^, ^4^

In this retrospective case-control study, we determined the prevalence of AAs and CRC in individuals 20–49 years old undergoing early screening colonoscopy due to a family history of advanced polyps and compared these data to patients in a control sample of the same age group without a family history who were undergoing their first colonoscopy for abdominal pain or altered bowel habits. We hypothesized that patients with an FDR of AA have an increased risk of AA compared with those without such a family history.

## Materials and Methods

**Figure 1:**
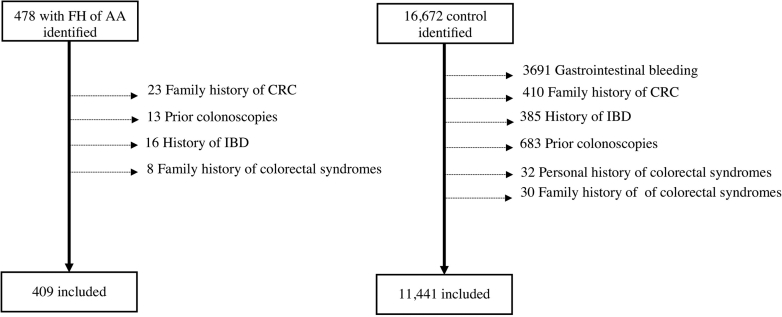
Study population with inclusion and exclusion criteria. IBD, inflammatory bowel disease.

To evaluate the performance of this guideline in a community setting, we conducted a single-center, retrospective, case-control study at a large community gastroenterology practice to compare the prevalence of AA and CRC in individuals undergoing early screening colonoscopy due to an FDR with an AA (known as the “FH” [family history] group) to average-risk individuals without a family history (known as the “control” group) between 2015 and 2023. The study was approved by the institutional review board of Providence Health. We identified 478 patients who underwent their index screening colonoscopy due to a family history of AA and 16,672 patients without a family history who underwent their first colonoscopy for evaluation of abdominal pain or bowel changes during the same period. We calculated that, with roughly 10,000 control patients available in our database, collecting data on 400 FH patients would provide greater than 80% power to detect a doubling of AA rate in FH relative to the control, assuming that the control rate was between 3% and 10% (minimum power = 82% to detect a difference between rates of 3% and 6% in a 2-sided test of proportions with alpha = 0.05). Individuals with a personal or family history of CRC or colorectal syndromes, personal history of inflammatory bowel disease, gastrointestinal bleeding, or prior colonoscopy were excluded (Figure 1). A total of 409 FH and 11,441 control patients were included in the study. Baseline characteristics are described in Table 1.

**Table 1:** Baseline Characteristics of the Study Population

Characteristics	FH (n = 409)	Control (n = 11,441)	*P* value
Age, median (IQR)	43 [40, 46]	44 [36, 47]	.9
Age, group, n (%)			<.001
20–39	96 (23.5)	3845 (33.6)	
40–44	149 (36.6)	2063 (18.0)	
45–49	164 (40.1)	5533 (48.4)	
Sex, n (%)			.4
Male	177 (43.3)	4712 (41.2)	
Female	232 (56.7)	6729 (58.1)	

The age groups (20–39, 40–44, and 45–49 y) represent the distribution of participants across predefined age categories. *P* values obtained from Mann-Whitney test (median age) or chi-square tests (age and sex groups).

FH, patients with a first-degree relative with a history of advanced adenomas; IQR, interquartile range.

Colonoscopy and accompanying pathology reports were reviewed for all patients included in the study. AAs were defined as adenomas with features of size ≥10 mm, villous characteristics, or high-grade dysplasia. The primary outcome was the prevalence of AA. The secondary outcome was rates of CRC. Patients were divided into 3 age groups of 20–39, 40–45, and 45–49 years old for statistical analysis. Comparisons of variable distributions between study groups were made with chi-squared tests of proportions or Mann-Whitney tests of numeric values. We used logistic regression to test the association of study group with outcomes after adjustment for patient sex (in age-restricted models) or sex, age group, and sex by age group interaction in analyses of all patients. All statistical analyses were conducted using R v.4.4.3 software.

## Results

**Figure 2:**
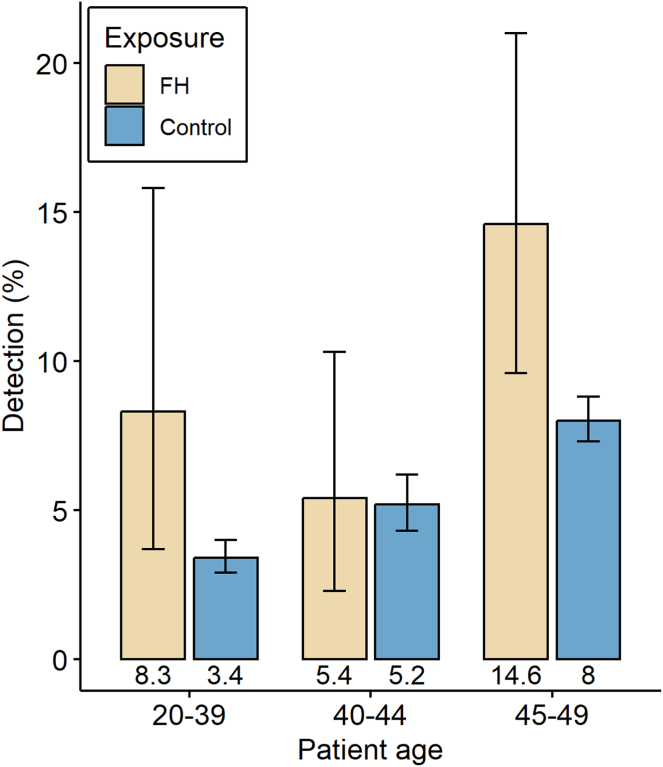
Detection rate of advanced adenomas in patients with family history (FH) and control group in a community practice. Bars show sample proportions with binomial 95% confidence intervals.

AAs were found in n = 40 (9.8%) FH patients and n = 682 in control patients (6.0%; *P* < .001 for difference between groups, by chi-squared test). The prevalence of AA in 20- to 39-year-old individuals in the FH group was 8.3% compared to 3.4% in the control group in the same age group (*P* < .012 by chi-squared test). The prevalence in 40- to 45-year-old individuals was similar between groups (5.4% and 5.2%, nonsignificant). In the 45–49 age group, the AA was 14.6% in the FH group and 8.0% in the control group (*P* < .001) (Figure 2). The overall adenoma detection rate was 50% in the FH group and 47.4% in the control group. No cases of CRC were identified in the FH group despite the inclusion of 409 individuals, whereas colon cancer was found in 0.36% of controls (n = 5, nonsignificant).

Logistic regression indicates similar increases in the rate of AA with a family history, after adjustment for interacting effects of patient sex and age group (odds ratio [OR] = 1.76; 95% confidence interval [CI] = 1.23–2.43; *P* < .001). When associations with family history were allowed to vary by age, while still adjusting for patient sex, we found significant increases in risk for the FH group in patients aged 20–34 (OR = 2.66; 95% CI = 1.17–5.29; *P* < .01) and 46–49 (OR = 1.96; 95% CI = 1.23–3.00; *P* < .003), but not in ages 40–44 (OR = 1.03; 95% CI = 0.46–2.04; *P* < .9) (Table 2).

**Table 2:** Prevalence of Advanced Adenomas in Patients With a Family History by Age

Age	FH, n (%) (n = 409)	Control, n (%) (n = 11,441)	OR (95% CI)	*P* value
20–39	8/96 (8.3)	131/3845 (3.4)	2.66 (1.17–5.29)	.01
40–44	8/149 (5.4)	107/2063 (5.2)	1.03 (0.46–2.04)	.9
45–49	24/164 (14.6)	444/5533 (8.0)	1.96 (1.23–3.00)	.003

This shows prevalence by age and study group, with odds ratio for advanced adenoma in FH relative to control and associated *P* values obtained from logistic regression adjusted for patient sex.

## Conclusion

Our study demonstrated that a family history of AA was associated with increased odds of AA, and the risk was nearly threefold higher in individuals of younger age. The current national guideline on screening in patients with a family history of advanced polyps is a conditional recommendation, with several limitations. One prospective study from Hong Kong found that siblings of individuals with AA had 6-fold increased odds of developing AA compared to siblings without a family history, however, the generalizability to a younger US population is limited by a lack of young patients in this study (only 7.5% of the study population was under the age of 50), low population screening rates, and a low adenoma detection rate of 15%.^5^

Our study has several strengths including the inclusion of individuals younger than 40 years old who are the target screening population for current guidelines and a high overall adenoma detection rate of 47.5%. Our retrospective study design also had several limitations. Despite the increasing access to electronic medical records, patient recall bias could skew the results, as the study authors did not have access to the family members’ medical records. Additionally, we used symptomatic patients as our control group, which could introduce confounding variables to endoscopic findings. Our study did not distinguish between advanced sessile serrated lesions and other advanced lesions, which may have different risk characteristics.^6^

While the US Multi-Society guidelines recommend colonoscopy at age 40 or 10 years before FDR was diagnosed, competing guidelines from the US Preventative Services Task Force and National Comprehensive Cancer Network do not recommend screening before age 40.^4^ Our study reinforces that screening this high-risk group of individuals may potentially reduce the burden of CRC. As screening practices have evolved with higher baseline adenoma detection rates and initiation of average-risk screening at age 45, this study and others underscore the need for prospective studies to evaluate the effectiveness of these competing recommendations.^7^
